# Individual differences in empathy are associated with apathy-motivation

**DOI:** 10.1038/s41598-017-17415-w

**Published:** 2017-12-11

**Authors:** Patricia L. Lockwood, Yuen-Siang Ang, Masud Husain, Molly J. Crockett

**Affiliations:** 1 Department of Experimental Psychology, University of Oxford, Oxford, OX1 3PH United Kingdom; 2 Wellcome Centre for Integrative Neuroimaging, Department of Experimental Psychology, University of Oxford, Oxford, United Kingdom; 3 Nuffield Department of Clinical Neurosciences, University of Oxford, Oxford, OX3 9DU United Kingdom; 4 Department of Psychology, Yale University, New Haven, CT 06511 USA

## Abstract

Empathy - the capacity to understand and resonate with the experiences of other people - is considered an essential aspect of social cognition. However, although empathy is often thought to be automatic, recent theories have argued that there is a key role for motivation in modulating empathic experiences. Here we administered self-report measures of empathy and apathy-motivation to a large sample of healthy people (n = 378) to test whether people who are more empathic are also more motivated. We then sought to replicate our findings in an independent sample (n = 198) that also completed a behavioural task to measure state affective empathy and emotion recognition. Cognitive empathy was associated with higher levels of motivation generally across behavioural, social and emotional domains. In contrast, affective empathy was associated with lower levels of behavioural motivation, but higher levels of emotional motivation. Factor analyses showed that empathy and apathy are distinct constructs, but that affective empathy and emotional motivation are underpinned by the same latent factor. These results have potentially important clinical applications for disorders associated with reduced empathy and motivation as well as the understanding of these processes in healthy people.

## Introduction

Empathy – the capacity to understand and resonate with the experiences of other people – is considered essential for navigating meaningful social interactions and is closely linked to prosocial behaviour^1–7^. Although there is a lack of consensus as to its precise definition, most theorists agree that empathy comprises a cognitive and an affective component that are correlated but also partially dissociable^3–8^. Cognitive aspects of empathy relate to the capacity for taking another individual’s perspective, commonly known as mentalising or theory of mind^3,9^. In contrast, affective empathy is understood as an affective state, caused by sharing the emotions of another person through observation or imagination of their experience^7,10^. Although the observer’s emotional state is isomorphic with the other person’s state, the observer is aware that the other person is the source of their state^3,10^.

Several disciplines of research, including philosophy, ethology, psychology and neuroscience, have treated empathy as an automatic process (for a review, see^11–13^). However, recent work has argued that this presumption could be unfounded^11–13^. Instead, although empathy can be automatic, there is also a crucial role for motivation in modulating empathetic experiences^11–13^. For instance, empathy is reduced for outgroup compared to ingroup members^14–18^ suggesting that empathy is not necessarily automatic, but occurs when people are motivated to empathise. Similarly, empathy is less apparent in response to the suffering of large groups than to small groups or individual people, again suggesting a lack of automaticity^19–21^. Importantly, many of these studies directly manipulate people’s motivation to empathize. There are also several direct demonstrations that increasing people’s motivation to empathize can increase state empathy. For example, activating in-group norms leads to stronger empathy for out-group members^22^ and observing empathic responses to vignettes can shift empathic feelings and decisions to donate to charity^23^. In addition, experimentally inducing a malleable theory of empathy, i.e. the idea that empathy can be developed rather than being fixed, leads to higher state empathy^24^.

More broadly, theoretical frameworks of motivated empathy highlight that there is a tension between empathizing with others and the potential deterrents to doing so, thus linking empathy to motivation. For example, empathizing with others may have benefits of positive affect, affiliation, and social desirability but also costs of cognitive effort, suffering, material costs, and interference with competition^11–13^. Similarly, theoretical frameworks of motivation highlight that the willingness to exert effort is based on an evaluation of the costs and benefits of performing an action (e.g.^25–28^). Therefore, it could be hypothesized that individual differences in motivation are related to individual differences in empathy. That is, those who are more empathic might also be more motivated and vice versa. Such a finding could have important implications for motivated empathy frameworks. It would suggest that empathy and motivation are not just associated in certain contexts, or when directly manipulated, but that empathy is closely interlinked with motivation.

Theories of human apathy – or loss of motivation – have proposed that, like empathy, motivation is comprised of correlated but distinct dimensions^28–31^. Pathological apathy in patients with brain disorders may include impairments in action initiation and emotional affect^32^. Consistent with the Research Domains and Criteria (RDoC) approach to psychiatric and neurological conditions, it is now increasingly accepted that apathy is a dimensional construct prevalent to varying degrees also in healthy people^29,31^. In line with this approach, a recent study identified three factors of apathy-motivation that could reliably be distinguished in healthy people^29^. These factors were *behavioural activation*, which was related to the tendency to initiate actions and included items such as “I don’t like to laze around”; *social motivation* that characterised the tendency to engage in social interactions and included items such as “I suggest activities for me and my friends to do”; and *emotional sensitivity* that captured the tendency to experience emotional affect and included items such as “I feel awful if I say something insensitive”.

The emotional component of apathy appears to overlap with some conceptualisations of affective empathy, where affective empathy is defined as emotional/affective resonance between self and other^3,7^. However, affective empathy also involves self/other distinction^3,7^ whereas emotional apathy, as defined here, largely focuses on emotional reactivity in oneself. Importantly, an account of the relationship between empathy and apathy needs to take into account the multidimensional nature of *both* constructs. This is so far lacking in the current literature.

Empathy and motivation may also overlap in clinical disorders. Reduced empathy and increased apathy are common co-occurring symptoms in both frontal temporal dementia (FTD) and amyotrophic lateral sclerosis (ALS) suggesting shared neurobiological correlates^33–35^. Recent accounts of psychopathy, a disorder associated with profound reductions in empathy, have also suggested a core role for motivation. Meffert and colleagues^36^ found that when criminal psychopaths were explicitly instructed to ‘empathise’, reductions in neural responses to others experiences that are commonly seen^37,38^ were alleviated^36^. These results suggest that psychopaths may have the capacity for empathy, but are not normally motivated to engage in empathising.

There is also evidence for associations between empathy and motivation in the typical population. The rate at which people discount or ‘devalue’ rewards by effort for others compared to self, a measure of prosocial motivation, correlates with individual differences in subclinical psychopathic traits^39^. It may also be that some dimensions of empathy are motivated but others are not. For example, individuals higher in cognitive empathy, but not affective empathy, learn faster which actions will result in rewards for others, suggesting that individuals high in cognitive empathy have increased prosocial motivation^2^. These findings lend further support to the idea that aspects of empathy may have a motivational component and that characterising these associations could have important clinical consequences. However, to our knowledge, the overlap and potential dissociations between aspects of empathy and apathy are currently unknown.

Here, we administered self-report measures of empathy and apathy-motivation to a sample of 378 healthy people and subsequently a separate sample of 198 healthy individuals with an aim to replicate our findings. This second sample also completed a behavioural task with two parts designed to assess *state* affective empathy and emotion recognition ability. We hypothesized that self-report measures of cognitive and affective empathy would be positively associated with self-report measures of motivation. We also hypothesized that performance on the state affective empathy and emotion recognition task would be correlated with self-reported empathy and emotional apathy.

## Results

### Overlap and distinction between empathy and apathy domains

We first used multiple regression analyses to examine unique associations between self-report measures of apathy, using the Apathy-Motivation Index^29^, and empathy, using the Questionnaire of Cognitive and Affective Empathy^40^ in a large sample of healthy adults (n = 378) (see Table S3 for bivariate correlations and associations with all empathy sub-subscales). In all regression analyses, we first controlled for age, given that age was correlated with apathy (see Supplemental results). At the second stage, affective and cognitive empathy were simultaneously added to examine distinct or similar associations with each apathy domain.

**Figure 1:**
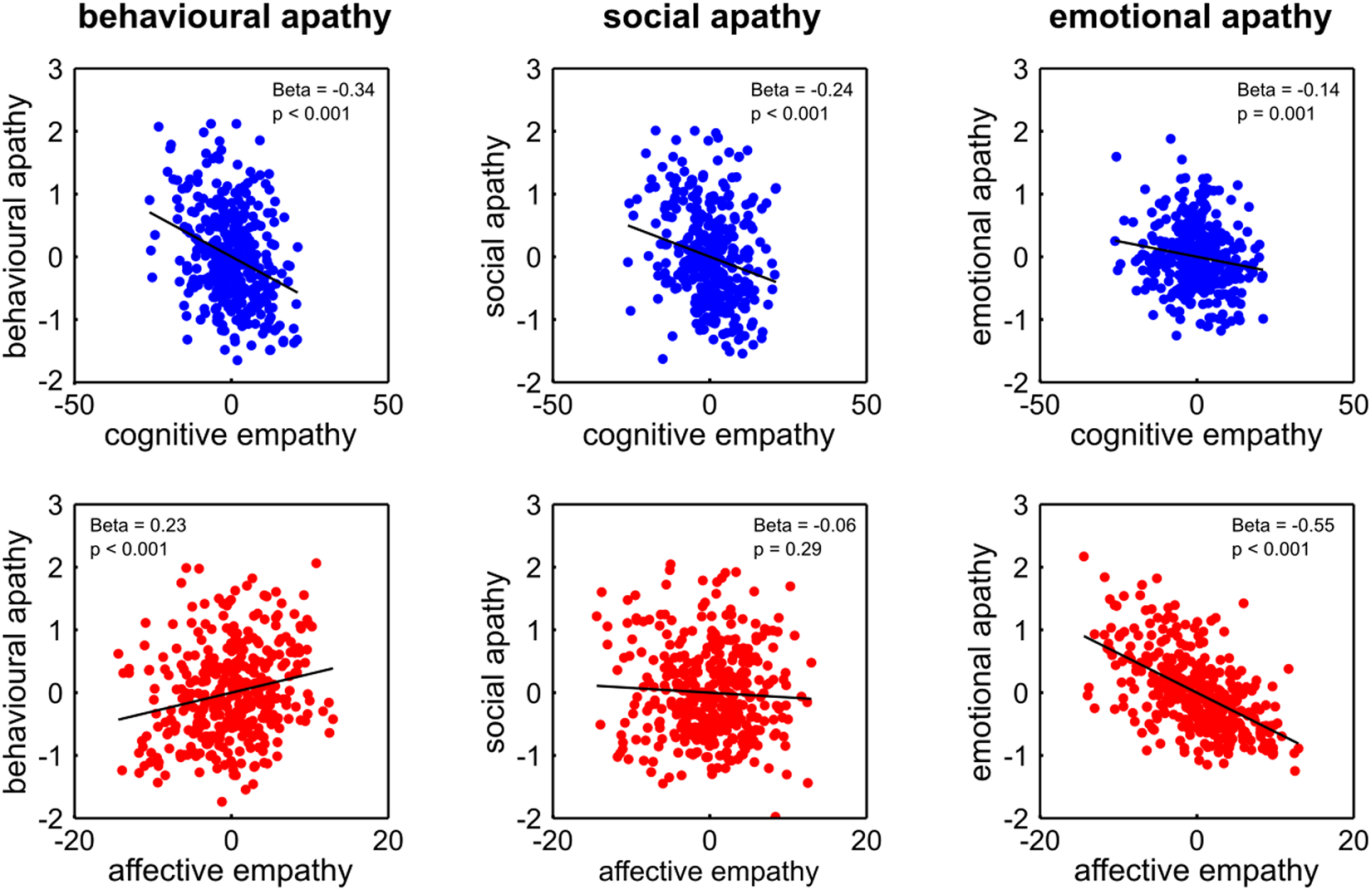
Relationships between different domains of empathy and apathy. Partial correlation plots from multiple regression models showing how unique variance associated with cognitive and affective empathy is associated with different domains of apathy-motivation (behavioural, social and emotional).

The first model predicted levels of behavioural apathy and showed that, intriguingly, affective and cognitive empathy exerted significant but opposing associations with behavioural apathy indicating a ‘suppressor effect’^41^ (Fig. 1). Specifically, while higher levels of *cognitive empathy* negatively predicted behavioural apathy, higher levels of *affective empathy* positively predicted behavioural apathy. Therefore, those high in cognitive empathy were behaviourally motivated whilst those high in affective empathy were behaviourally apathetic.

The second model predicted levels of social apathy and showed that only *cognitive empathy* was a significant negative predictor of *social apathy*, with those higher in cognitive empathy being more socially motivated (Fig. 1). For the third model that predicted levels of emotional apathy, both *affective* and *cognitive empathy* were significant negative predictors, showing that these two types of empathy were linked to higher levels of emotional motivation and lower levels of emotional apathy (Fig. 1). Overall, these findings suggest that people high in cognitive empathy are more motivated across behavioural, social and emotional domains, whereas those high in affective empathy may be less behaviourally motivated, but are more emotionally motivated.

### Replication of correlations and regressions between apathy and empathy domains

**Figure 2:**
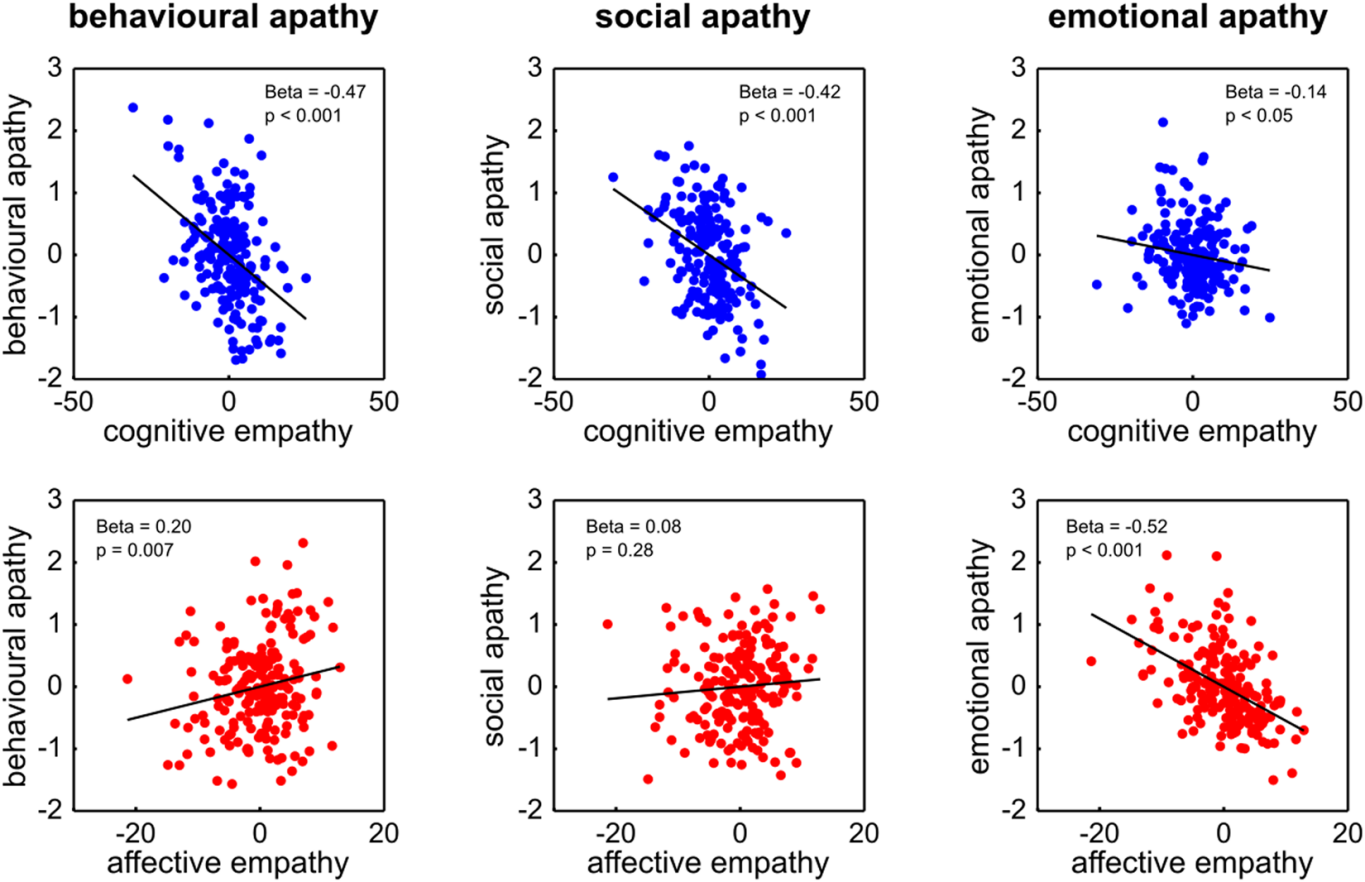
Relationships between domains of empathy and apathy in Sample 2. Replication of partial correlations from multiple regression models showing how unique variance associated with cognitive and affective empathy is associated with different domains of apathy-motivation (behavioural, social and emotional).

We next sought to replicate our findings in an independent sample of healthy people (n = 198) who also completed a task to assess state affective empathy and emotion recognition (see Experimental Procedures for participant demographics). We found that all previously reported multiple regression analyses with empathy and apathy replicated the findings from the first study (Fig. 2, See Table S4 for bivariate associations and associations with all empathy sub-subscales).

### Associations between state affective empathy, emotion recognition, empathy and apathy

Next, we examined how empathy and apathy were correlated with a task that assessed *state affective empathy* and *emotion recognition*. For the state affective empathy measure, which was based on previous studies using a similar approach^8,42–44^, participants first rated their emotional response to videos depicting different emotional expressions (happy, sad, fear, anger, disgust) on a 9-point likert scale from positive to negative (see Experimental Procedures for full details). Then participants judged the emotional expression of each agent in a forced choice that presented the different emotion labels (happy, sad, fear, anger, disgust, neutral) in a randomised order. We created a composite score for the state affective empathy task (combining ratings for happy, sad, disgust, fear and anger ratings) and a composite score for the emotion recognition task (combining percentage accuracy for sad, disgust, fear and anger trials only, because 80% of participants performed at ceiling for identifying happy faces). These two scores were then correlated with self-reported empathy and apathy.

### Correlations and unique associations between apathy, empathy and state affective empathy

We found that trait *cognitive empathy and affective empathy* were both significantly positively associated with *state* affective empathy (*r* = 0.23, *p* = 0.002 *and r* = 0.34, *p* < *0*.001). However, after controlling for shared variance between cognitive and affective empathy, only *trait* affective empathy positively predicted *state* affective empathy (*β* = 0.29, *p* < 0.001).

Self-reported behavioural, social and emotional apathy were all significantly negatively associated with state affective empathy (Behavioural apathy *r* = −0.16, *p* = 0.03; Social apathy *r* = −0.29, *p* < 0.001; Emotional apathy *r* = −0.30, *p* < 0.001). After controlling for shared variance using regression analyses, social (*β* = −0.23, *p* = 0.003) and emotional apathy (*β* = −0.25, *p* < 0.001) predicted state empathy, but behavioural apathy did not (*β* = −0.03, *p* = 0.71). Overall these findings suggest that both trait empathy components are associated with greater state affective empathy, and that social and emotional motivation are also associated with greater state affective empathy.

### Correlations and unique associations between apathy, empathy and emotion recognition

Cognitive and affective empathy were positively correlated with emotion recognition ability (*r* = 0.17, *p* = 0.03 and *r* = 0.15, *p* = 0.03). Regression analyses showed that shared variance predicted greater emotion recognition ability, as neither empathy component was positively associated with emotion recognition after controlling for shared variance between cognitive and affective empathy (all *β* < *0.12, all p’s* > *0.12)*.

Emotional apathy was negatively correlated with emotion recognition (*r* = −0.19, *p* = 0.021), but social and behavioural apathy were not (all *r’s* < −0.08, all *p’s* > 0.45). Finally, when controlling for shared variance, only emotional apathy was associated with significantly poorer emotion recognition (*β* = −0.20, *p* = 0.006). Together these findings point to higher emotion recognition abilities in those with greater affective and cognitive empathy, and in those lower in emotional apathy.

### Factor analysis supports overlap and distinction between apathy and empathy

To further investigate the overlap and distinction between components of apathy and empathy, we conducted an exploratory factor analysis on all items of the empathy and apathy-motivation measures. A five-factor structure was found to be the most parsimonious account of the data. This model had good fit indices (Root Mean Square Error of Approximation, RMSEA = 0.059 with 90% CI of 0.056 – 0.063; Standardised Root Mean Square Residuals, SRMR = 0.04), and was supported by the scree plot^45^, which showed a plateau in eigenvalues after five factors.

**Figure 3:**
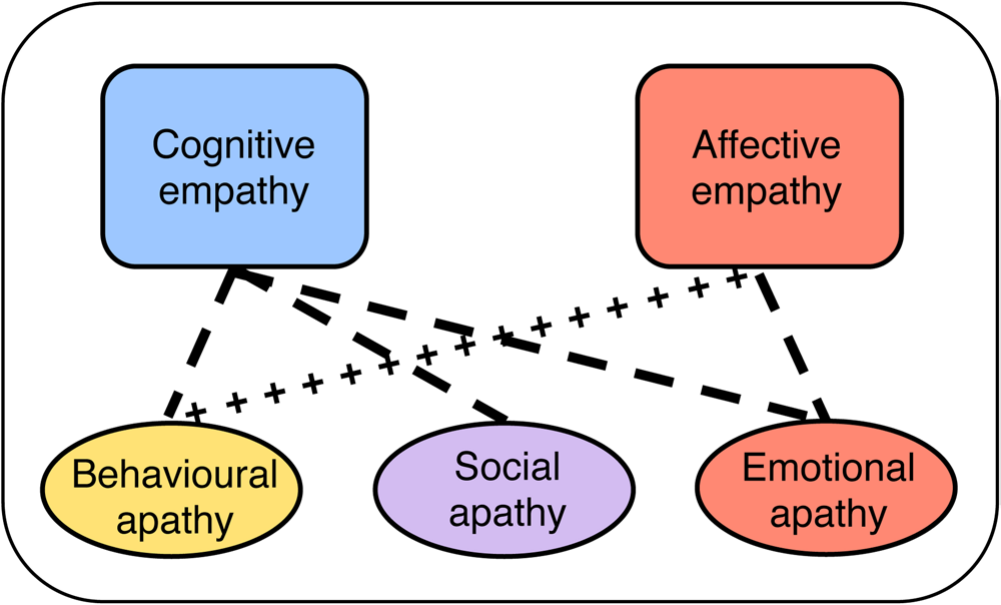
Schematic of associations between empathy and apathy-motivation. People higher in cognitive empathy are less behaviourally, socially and emotionally apathetic, i.e. more motivated in general. However, those higher in affective empathy are more behaviourally apathetic, but less emotionally apathetic. Factor analyses showed that lack of affective empathy and emotional apathy are underpinned by the same latent factor (red box and oval). Dashed lines represent negative associations and crossed lines represent positive associations from multiple regression analyses of unique variance associated with empathy and apathy-motivation.

Items that loaded most strongly onto Factor 1 comprised those from the emotional apathy subscale and affective empathy subscale (Table 1, italic), indicating that affective empathy and emotional motivation are overlapping domains supported by the same latent factor. The other four factors were associated with distinct latent factors. Specifically, Factors 2–5 characterised behavioural apathy, social apathy, cognitive empathy via online simulation, and cognitive empathy via perspective taking. Similar results were obtained using the replication sample in Study 2 (Table 1, underline). These findings suggest that apathy and empathy are partially dissociable constructs that may overlap in the affective domain (Fig. 3, red).

**Table 1:** Item loadings support a 5-factor structure of items related to empathy and apathy-motivation.

Item & subscale		Sample 1 (n = 378)					Sample 2 (n = 198)				
		Factor					Factor				
		**1**	**2**	**3**	**4**	**5**	**1**	2	3	4	5
**AMI 1**	**ES**	***0.48***	0.05	0.17	0.00	−0.08	0.56	−0.08	0.10	−0.07	0.03
**AMI 6**	**ES**	***0.36***	0.17	−0.04	0.06	−0.06	0.45	0.00	−0.02	−0.12	−0.10
**AMI 7**	**ES**	***0.40***	−0.12	0.09	0.07	0.02	0.40	0.34	0.10	0.02	−0.10
**AMI13**	**ES**	***0.51***	−0.07	−0.04	0.31	−0.10	0.53	0.06	−0.11	0.24	−0.12
**AMI16**	**ES**	***0.36***	−0.12	0.04	0.24	−0.09	0.53	0.10	0.06	0.00	0.09
**AMI18**	**ES**	***0.55***	−0.06	−0.08	0.34	−0.10	0.45	0.14	0.09	0.28	−0.11
**QCAE 2**	**AE**	*−* ***0.40***	0.05	0.03	0.13	−0.03	0.40	0.01	−0.20	−0.07	0.10
**QCAE 7**	**AE**	*−* ***0.44***	−0.02	−0.25	−0.05	−0.11	−0.49	0.13	−0.12	−0.13	−0.09
**QCAE 8**	**AE**	*−* ***0.63***	−0.08	0.21	0.14	0.05	−0.70	0.26	−0.05	−0.09	0.13
**QCAE 9**	**AE**	*−* ***0.65***	−0.16	−0.00	0.22	−0.20	−0.70	0.01	−0.02	0.15	0.04
**QCAE 10**	**AE**	*−* ***0.69***	0.01	−0.05	−0.04	−0.01	−0.65	−0.04	0.06	−0.19	0.03
**QCAE 11**	**AE**	*−* ***0.51***	−0.06	−0.06	0.06	−0.08	−0.60	−0.18	0.17	0.01	0.02
**QCAE 12**	**AE**	*−* ***0.63***	0.05	0.07	−0.12	0.06	−0.57	0.01	0.12	−0.11	−0.01
**QCAE 13**	**AE**	*−* ***0.53***	0.02	−0.10	0.09	0.02	−0.66	−0.00	−0.14	0.20	0.03
**QCAE 14**	**AE**	*−* ***0.64***	−0.00	0.12	0.05	0.05	−0.76	0.13	−0.03	−0.04	0.08
**QCAE 17**	**AE**	−0.11	0.03	0.05	−0.25	−0.14	0.16	−0.10	−0.18	0.36	0.21
**QCAE 23**	**AE**	−0.10	0.01	−0.11	−0.21	*−* ***0.40***	−0.29	−0.20	−0.13	0.09	−0.40
**QCAE 29**	**AE**	*−* ***0.35***	0.06	−0.02	0.02	−0.06	0.40	−0.07	−0.16	−0.07	0.17
**AMI 5**	**BA**	−0.32	−0.23	0.27	0.01	0.13	−0.24	0.56	0.19	0.09	−0.08
**AMI 9**	**BA**	−0.02	*−* ***0.67***	0.11	−0.13	0.06	−0.17	0.60	0.15	−0.11	0.12
**AMI10**	**BA**	−0.07	*−* ***0.47***	0.11	0.01	−0.04	0.02	0.57	−0.01	0.04	−0.12
**AMI11**	**BA**	0.04	*−* ***0.81***	−0.12	−0.03	−0.01	−0.07	0.70	−0.05	0.08	0.10
**AMI12**	**BA**	0.04	*−* ***0.80***	−0.06	−0.08	0.03	−0.02	0.85	0.01	−0.03	−0.02
**AMI15**	**BA**	−0.11	*−* ***0.71***	−0.08	0.10	−0.08	0.03	0.78	−0.12	−0.04	0.03
**AMI 2**	**SM**	−0.01	0.10	***0.71***	−0.01	0.01	−0.03	0.01	0.58	0.05	0.01
**AMI 3**	**SM**	−0.13	0.14	***0.82***	0.04	−0.16	−0.05	0.03	0.71	0.21	−0.12
**AMI 4**	**SM**	0.15	−0.14	***0.52***	−0.07	−0.03	0.05	0.06	0.60	−0.09	0.11
**AMI 8**	**SM**	0.09	−0.07	***0.49***	−0.09	−0.06	0.01	−0.12	0.58	−0.05	0.13
**AMI14**	**SM**	−0.02	0.01	***0.71***	0.02	0.03	0.14	0.18	0.29	−0.17	0.22
**AMI17**	**SM**	0.03	−0.05	***0.36***	0.05	0.05	0.07	0.39	0.24	0.03	0.07
**QCAE 1**	**OS**	0.04	−0.05	−0.10	*−* ***0.59***	−0.04	−0.00	−0.07	−0.21	0.46	0.28
**QCAE 3**	**OS**	−0.03	−0.07	0.10	*−* ***0.66***	−0.04	−0.05	−0.07	−0.02	−0.59	−0.02
**QCAE 4**	**OS**	−0.08	0.05	−0.03	*−* ***0.65***	−0.05	−0.08	−0.04	−0.12	−0.56	−0.02
**QCAE 5**	**OS**	0.08	−0.07	−0.02	*−* ***0.82***	−0.04	−0.09	0.01	−0.17	−0.78	0.11
**QCAE 6**	**OS**	−0.02	−0.02	0.02	*−* ***0.80***	0.10	0.09	0.08	−0.09	−0.81	0.04
**QCAE 18**	**OS**	0.03	−0.08	−0.10	*−* ***0.59***	−0.22	0.01	0.01	0.02	−0.63	−0.26
**QCAE 28**	**OS**	0.02	0.01	0.01	*−* ***0.42***	−0.18	−0.01	−0.22	−0.01	−0.32	−0.04
**QCAE 30**	**OS**	−0.13	0.07	0.08	*−* ***0.59***	−0.07	−0.13	−0.10	0.05	−0.54	−0.04
**QCAE 31**	**OS**	−0.34	0.01	0.02	−0.31	−0.07	−0.32	0.03	−0.02	−0.25	−0.19
**QCAE 15**	**PT**	−0.07	0.03	−0.10	0.11	*−* ***0.61***	0.11	−0.02	−0.14	0.01	−0.70
**QCAE 16**	**PT**	0.07	−0.09	−0.01	0.07	*−* ***0.78***	0.01	−0.05	−0.05	−0.00	−0.72
**QCAE 19**	**PT**	0.07	−0.02	−0.05	−0.22	*−* ***0.61***	0.09	0.09	−0.06	−0.16	−0.75
**QCAE 20**	**PT**	−0.04	−0.03	0.05	0.03	*−* ***0.81***	−0.09	0.17	−0.11	−0.08	−0.71
**QCAE 21**	**PT**	−0.09	−0.01	−0.15	−0.18	*−* ***0.51***	−0.21	−0.00	−0.29	−0.20	−0.32
**QCAE 22**	**PT**	0.05	−0.04	0.14	−0.06	*−* ***0.76***	0.06	−0.22	0.28	0.06	−0.73
**QCAE 24**	**PT**	−0.09	0.10	0.22	0.01	*−* ***0.72***	0.05	0.01	0.03	−0.10	−0.69
**QCAE 25**	**PT**	−0.03	0.06	−0.05	−0.13	*−* ***0.58***	0.05	−0.02	−0.15	0.00	−0.66
**QCAE 26**	**PT**	0.04	0.02	0.04	0.03	*−* ***0.77***	0.02	−0.01	−0.05	−0.04	−0.72
**QCAE 27**	**PT**	0.10	0.06	−0.02	−0.04	*−* ***0.65***	0.12	−0.07	0.07	0.07	−0.77

The AMI (Apathy-Motivation Index) is an 18-item self-report measure of apathy that comprises three subscales: (1) ES: emotional sensitivity (emotional apathy, as all items reverse scored), (2) SM: social motivation (social apathy, as all items reverse scored), and (3) BA: behavioural activation (behavioural apathy, as all items reverse scored). The QCAE (Questionnaire of Cognitive and Affective Empathy) is a 31-item self-report measure of empathy. It contains two main subscales (1) AE: affective empathy, and (2) cognitive empathy that may be further subdivided into either (2a) OS: online simulation, or (2b) PT: perspective taking. Items with loadings >0.35 were highlighted in bold italic (sample 1) and underline (sample 2). Factor 1 is a general emotional construct comprising items from AMI ES and QCAE AE. Factors 2–5 mainly contained items from AMI BA, AMI SM, QCAE OS, and QCAE PT respectively.

## Discussion

Although empathy is often considered to be an automatic process, recent theories have argued that there is a key role for motivation in modulating empathic experience^11–13,46^. Experimental findings suggest that those who are more empathic may also be more motivated^2,39^, and increasing people’s motivation to empathize can increase state empathy^22–24^. Here we examined whether and how empathy and apathy-motivation are associated in two independent samples of healthy people using self-report measures, and in the second sample, using a task to assess state affective empathy and emotion recognition. Across both samples, those who were higher in cognitive empathy reported higher levels of behavioural, social and emotional motivation. When accounting for shared variance between cognitive and affective empathy, those higher in affective empathy were in fact *less behaviourally motivated* but *more emotionally motivated* (Fig. 3). These findings were replicated across two independent samples.

Trait affective empathy, but not cognitive empathy, was correlated with higher ‘state’ affective empathy after accounting for shared variance. Social apathy and emotional apathy, but not behavioural apathy, were negatively associated with state affective empathy. Shared variance between affective and cognitive empathy predicted greater emotion recognition abilities. Higher levels of emotional apathy were related to poorer emotion recognition abilities, but higher levels of behavioural and social apathy were not. Finally, factor analyses showed that dimensions of empathy and apathy are generally distinct. However, affective empathy and emotional motivation were underpinned by the same latent factor.

Overall these findings support the idea that empathy is indeed closely linked to motivation^2,13,36^ but also that it is essential to consider the multifaceted nature of both constructs. Those with high levels of cognitive empathy have higher levels of motivation in behavioural, social and emotional domains, whereas those high in affective empathy have lower levels of behavioural motivation whilst at the same time more emotional motivation. Intriguingly, both types of empathy have been linked to higher levels of self-reported prosocial tendencies^47^. However, here we show that, surprisingly, trait affective empathy is not consistently associated with higher levels of social motivation. This finding is in line with the results of a recent study which shows that those higher in trait cognitive empathy – but not affective empathy – learn faster which of their actions will result in rewards for others, an index of prosocial motivation^2^. Together these findings suggest a potentially greater role for cognitive empathy than affective empathy in behaviours related to social motivation. However, this hypothesis would need to be tested in further studies with a broad range of socially motivated behaviours.

We also observed differing patterns of associations between empathy and apathy, and emotion recognition abilities. Whilst both affective and cognitive empathy were correlated with higher accuracy in recognising emotions (e.g.^48–50^), only emotional apathy was correlated with reduced emotion recognition abilities. This suggests that despite the overlap between empathy and apathy, emotion recognition, a key social cognitive ability, is only associated with empathy and the emotional aspects of apathy, and not with behavioural or social motivation, at least with the present measures. There may also be some differential associations between how behavioural apathy relates to state versus trait affective empathy. Across two independent samples we found that behavioral apathy was positively associated with trait affective empathy after controlling for shared variance with trait cognitive empathy. However, for state affective empathy, we found a significant negative association with behavioural apathy in bivariate associations, that disappeared after controlling for shared variance with the other apathy domains. Further studies would be useful to probe the surprising positive association between trait affective empathy and behavioural apathy. Understanding this result further could help to inform current debates about the limits of empathy^13,46^. For example, if affective empathy were linked with emotionality but not actual behavior, this would suggest that the relationship between affective empathy and prosocial action is unclear. This would support findings from a recent study showing that emotion regulation moderates the association between affective empathy and prosocial behaviour, that is, affective empathy may not always translate into prosocial action^47^.

The focus of our study was to assess whether variability in different types of empathy was associated with apathy in behavioural, social or emotional domains. However, further studies would benefit from broadening this work to focus on more specific motivations, such as the motivation to help out-groups or to be prosocial, which may involve aspects of all three types of motivation. Although we found that affective empathy and emotional apathy are underpinned by the same latent construct, it is important to note that in bivariate associations shared variance between emotional apathy and affective empathy was less than 40%. In light of these findings, it would be interesting to test whether there are instances where affective empathy and emotional apathy can dissociate.

We focused our analyses on unique variance, as we were interested to examine unique contributions of different types of empathy to variability in apathy-motivation. Whilst in everyday life it is likely that both types of empathy would be co-activated, and indeed the correlation between the two subcomponents has been shown to be high in several studies^40,47^, there is also evidence that in the typical population^8,51^ and clinical disorders^52,53^ the two types of empathy can dissociate and/or can be independent. In the present study, we also observed unique contributions of affective and cognitive empathy to apathy, providing further support that although closely related the two types of empathy may dissociate in certain situations.

Factor analyses with items from the empathy and apathy measures revealed that affective empathy and emotional motivation are associated with the same underlying factor, which could have important implications scientifically. Researchers studying lack of affective empathy and emotional aspects of apathy may in fact be investigating a similar construct with a common basis and could therefore draw on the findings from these different fields. Items in the affective empathy measure assess emotional responses to others^40^, whereas items in the emotional apathy subscale assess mainly emotional sensitivity in general to both self and other^29^. The overlap between these processes points to a common domain of emotion that relates general emotional reactivity in oneself to the experience of emotion in response to others, in line with several theories of empathy^3,54^. This also supports recent studies showing that levels of harm aversion in self and for others are highly correlated^55^. Focusing on this common mechanism underlying emotional apathy and affective empathy may raise new questions in the study of motivated empathy.

Our finding that affective empathy and emotional motivation are underpinned by the same latent factor also has potentially important implications clinically. Changes in empathy and apathy are co-occurring clinical features of several neurological disorders including fronto-temporal dementia (FTD), Alzheimer’s disease (AD) and amyotrophic lateral sclerosis (ALS)^33–35^. However, clinically, empathy and apathy are each treated as unitary constructs. For example, the international consensus criteria for behavioural variant FTD lists apathy and early loss of sympathy or empathy as key symptoms, but does not distinguish subdomains within each of the two behavioural syndromes^35^. Moreover, there is initial evidence that subdomains of empathy might indeed be differentially affected in neurological disorders. Dermody and colleagues found that patients with FTD had impaired cognitive and affective empathy but that in AD only cognitive empathy was disrupted^8^. Further studies in clinical populations are essential for understanding the multidimensional nature of both constructs and whether diagnostic symptoms associated with empathy and apathy are primary or secondary to one another.

Frameworks of motivated empathy suggest that motivation modulates empathic outcomes, i.e. they are distinct processes but correlated with one another. Whilst our results cannot provide causal evidence in support of this hypothesis we do find support for a key premise of motivated empathy accounts that the two are linked. Crucially we provide greater clarity to their exact relationship at the trait level showing that although emotional motivation and affective empathy could be underpinned by the same latent factor, other aspects of motivation and empathy are distinct from one another. Moreover, whilst previous studies of motivated empathy have focused on the relationship between motivational and empathic states, to our knowledge no studies have investigated associations between these constructs at the trait level. Here we address an important piece of the broader theoretical question of motivated empathy by showing that apathy and empathy are linked at the trait level.

Overall, we provide evidence for a close relationship between aspects of empathy and apathy-motivation. Those who are high in cognitive empathy report that they are more motivated in general across behavioural, social and emotional domains, whereas those high in affective empathy are less behaviourally motivated but more emotionally motivated. However, when it comes to recognising emotional expressions, only empathy and emotional aspects of apathy are associated with greater emotion recognition ability. These findings could have potentially important clinical applications for disorders associated with reduced empathy and motivation as well as the understanding of these processes in healthy people.

## Methods

### Study 1 | Relationship between empathy and apathy

#### Participants

378 people (Age 16–74 (3 undisclosed), M = 30.5, SD = 11.2, 201 male, 177 female) were recruited via online adverts and Prolific Academic. The exclusion criteria were self-reported history of neurological or psychiatric disorders. All participants gave electronic informed consent and the study was approved by the University of Oxford ethics committee and carried out in accordance with the relevant guidelines and regulations.

#### Procedure

Participants completed two questionnaires to measure empathy and apathy.

The Apathy-Motivation Index (AMI^29^): Apathetic traits were measured using the Apathy-Motivation Index (AMI^29^). The AMI is an 18-item scale used to measure individual differences in apathy-motivation. Participants are asked to indicate their agreement with each item on a 5-point likert scale from 0–4. All items are reverse scored such that higher scores indicate higher apathy. The AMI consists of three subscales each representing a different domain of apathy: behavioural activation, “I don’t like to laze around”, (henceforth behavioural apathy), social motivation, “I suggest activities for me and my friends to do”, (henceforth social apathy) and emotional sensitivity, “I feel awful if I say something insensitive”, (henceforth emotional apathy). The development of the AMI followed a stringent set of procedures. First, a large sample (*n* = 505) of participants completed a set of items taken from existing apathy measures. These items were subjected to an exploratory factor analysis followed by a confirmatory factor analysis in an independent sample (*n* = 479). Test-retest reliability analyses showed good test re-test reliability. The AMI also correlated strongly with existing measures of apathy and depression, suggesting good convergent validity. Internal consistency analyses suggested good internal consistency (Cronbach’s *α* > 0.75 for all subscales), overall indicating that the AMI has good psychometric properties^29^. Moreover, there is evidence that variability in apathy as measures by the AMI relates to behavioural outcomes. The rate at which people devalue rewards by effort, an implicit behavioural index of apathy used in several studies (e.g.^39,56,57^) correlates with the behavioural apathy subscale of the AMI. The social apathy subscale of the AMI correlates with the willingness to put in effort for other people, a behavioural marker of social motivation^39^.

The Questionnaire of Cognitive and Affective Empathy (QCAE^40^): The QCAE, is a multidimensional empathy questionnaire devised to measure the ability to comprehend the emotions of another (cognitive empathy) as well as the ability to vicariously experience the emotional experience of others (affective empathy). We chose to use the QCAE as although the Interpersonal Reactivity Index (IRI)^58^ has been hugely influential in the field of empathy research, the IRI contains no specific measure of vicarious experience, only empathic concern (sympathy) and personal distress, and thus does not measure the conceptualisation of empathy adopted in the current manuscript and in the field more generally (e.g.^3,6,7^). In contrast, the QCAE was developed to assess the multidimensional nature of empathy, and to more closely reflect current definitions of empathy. In the development of the QCAE, two raters selected items from other commonly used empathy measures (e.g. Hogan Empathy Scale (HES^59^), Interpersonal Reactivity Index (IRI^58^), Balanced Emotional Empathy Scale (BEES^60^), and Empathy Quotient (EQ;^61^) if they were deemed to measure empathy. Items deemed to measure other processes (e.g. sympathy) were not included. These items were then subjected to an exploratory factor analysis to identify the underlying structure of associations, and then to a confirmatory factor analysis in a separate sample to confirm this underlying structure. Such an approach can be seen as a gold standard in questionnaire development and validation^62^. The cognitive empathy dimension comprises subscales measuring perspective-taking (e.g. “I am good at predicting how someone will feel”) and Online simulation (e.g. “Before criticizing somebody, I try to imagine how I would feel if I was in their place.”). The affective subscales assess emotion contagion (e.g. “People I am with have a strong influence on my mood”); peripheral responsivity (e.g. “I usually stay emotionally detached when watching a film”); and proximal responsivity (e.g. “I often get emotionally involved with my friends’ problems”). We focused our analysis on the subcomponents of affective and cognitive empathy, rather than separate subscales of perspective-taking, online simulation, emotional contagion, peripheral responsivity and proximal responsivity, in order to reduce the number of comparisons and because we had no specific hypothesis about how these sub-subscales might relate to subcomponents of apathy. Items were rated on a 4-point scale from “strongly disagree” to “strongly agree”. The QCAE has good validity and internal consistency^40^.

### Study 2 | Replication of associations between empathy and apathy

#### Participants

199 people (Age 20–63 (1 undisclosed), M = 34.3, SD = 10.7, 102 male, 96 female) were recruited via online adverts and Prolific Academic. The exclusion criteria were self-reported history of neurological or psychiatric disorders. One person was excluded after data collection for poor performance on the emotion recognition task (~20% accuracy across all emotions). All participants gave electronic informed consent and the study was approved by the University of Oxford ethics committee and carried out in accordance with the relevant guidelines and regulations.

#### Procedure

Participants again completed the apathy and empathy questionnaire measures and additionally completed a task with two parts. The first part assessed state affective empathy and the second part assessed emotion recognition.

Affective empathy and emotion recognition task: The state affective empathy task was an adapted version of the Self-Assessment Manikin Task^8,43,44,63^. Several studies have used variations of the Self-Assessment Manikin to assess basic affective resonance at the state level^8,42–44,63^. These studies have robustly shown replicable findings, such as that psychopathic traits are consistently associated with reduced affective resonance to emotional faces^8,43,44^ providing convergent validity that the SAM is a valid proxy measure of online or state affective empathy. Our modified version used videos of emotional expressions rather than static pictures, and a rating scale instead of a picture based manikin scale (as in^42^). Stimuli comprised of ~1000 ms video clips of six emotional expressions, Happy, Sad, Fearful, Angry, Disgusted and Neutral, edited for length from the Multimedia Understanding Group Facial Expression Database^64^. There were 6 female identities and 7 male identities that each expressed the 6 emotions (78 trials in total). For each identity/expression participants rated how they felt when on an 11-point scale (0–10) ranging from ‘extremely negative’ to ‘extremely positive’.

For our emotion recognition measure, we followed several other established paradigms by giving participants a forced choice task where they were required to label each emotion from a set of basic emotion labels^65^. There is evidence that a forced choice format is a valid way to assess emotion recognition performance^66^ and provides a quick and easy quantitative evaluation of answers^67^, although we acknowledge that there have been challenges to the use of forced choice paradigms^68^. The labels for the 6 emotions were presented in a randomised order. All identities and expressions were also presented in a randomised order.

### Data analyses – both studies

Bivariate correlations were corrected for multiple comparisons using Benjamini & Hochberg False Discovery Rate^69^. Corrected p-values are reported. Factor analyses were conducted using promax rotation in MPlus^70^.

### Data availability statement

All data generated or analysed during this study are included in this published article (and its Supplementary Information files).

## Electronic supplementary material

Supplementary Information

Supplementary dataset sample 1

Supplementary dataset sample 2

## References

[1] Lockwood PL (2016). The anatomy of empathy: Vicarious experience and disorders of social cognition. Behav. Brain Res..

[2] Lockwood PL, Apps MAJ, Valton V, Viding E, Roiser JP (2016). Neurocomputational mechanisms of prosocial learning and links to empathy. Proc. Natl. Acad. Sci..

[3] Bird G, Viding E (2014). The self to other model of empathy: Providing a new framework for understanding empathy impairments in psychopathy, autism, and alexithymia. Neurosci. Biobehav. Rev..

[4] Decety J, Jackson PL (2004). The functional architecture of human empathy. Behav. Cogn. Neurosci. Rev..

[5] Eisenberg N (2000). Emotion, regulation, and moral development. Annu. Rev. Psychol..

[6] Hoffman ML (2008). Empathy and prosocial behavior. Handb. Emot..

[7] Singer & Lamm C (2009). The social neuroscience of empathy. Ann. N. Y. Acad. Sci..

[8] Lockwood PL, Bird G, Bridge M, Viding E (2013). Dissecting empathy: high levels of psychopathic and autistic traits are characterized by difficulties in different social information processing domains. Front. Hum. Neurosci..

[9] Frith CD, Frith U (2006). The neural basis of mentalizing. Neuron.

[10] De Vignemont F, Singer T (2006). The empathic brain: how, when and why?. Trends Cogn. Sci..

[11] Cameron, D. *et al*. Empathy is hard work: People choose to avoid empathy because of its cognitive costs (2016). 10.1037/xge0000595 30998038

[12] Cameron, C. D., Inzlicht, M. & Cunningham, W. a. Deconstructing empathy: A motivational framework for the apparent limits of empathy. Retrieved from osf.io/preprints/psyarxiv/d99bp. *PsyArXiv* (2017).

[13] Zaki J (2014). Empathy: A Motivated Account. Psychol. Bull..

[14] Cikara M, Bruneau EG, Saxe RR (2011). Us and them intergroup failures of empathy. Curr. Dir. Psychol. Sci..

[15] Cikara M, Van Bavel JJ (2014). The Neuroscience of Intergroup Relations. Perspect. Psychol. Sci..

[16] Gutsell JN, Inzlicht M (2010). Empathy constrained: Prejudice predicts reduced mental simulation of actions during observation of outgroups. J. Exp. Soc. Psychol..

[17] Gutsell JN, Inzlicht M (2012). Intergroup differences in the sharing of emotive states: neural evidence of an empathy gap. Soc. Cogn. Affect. Neurosci..

[18] Levine M, Cassidy C, Brazier G, Reicher S (2002). Self-Categorization and Bystander Non-intervention: Two Experimental Studies1. J. Appl. Soc. Psychol..

[19] Cameron CD, Payne BK (2011). Escaping affect: how motivated emotion regulation creates insensitivity to mass suffering. J. Pers. Soc. Psychol..

[20] Dickert S, Kleber J, Peters E, Slovic P (2011). Numeracy as a precursor to pro-social behavior: The impact of numeracy and presentation format on the cognitive mechanisms underlying donation decisions. Judgm. Decis. Mak..

[21] Smith RW, Faro D, Burson KA (2013). More for the many: The influence of entitativity on charitable giving. J. Consum. Res..

[22] Tarrant M, Dazeley S, Cottom T (2009). Social categorization and empathy for outgroup members. Br. J. Soc. Psychol..

[23] Nook EC, Ong DC, Morelli SA, Mitchell JP, Zaki J (2016). Prosocial conformity: Prosocial norms generalize across behavior and empathy. Pers. Soc. Psychol. Bull..

[24] Schumann K, Zaki J, Dweck CS (2014). Addressing the empathy deficit: beliefs about the malleability of empathy predict effortful responses when empathy is challenging. J. Pers. Soc. Psychol..

[25] Phillips PEM, Walton ME, Jhou TC (2007). Calculating utility: preclinical evidence for cost-benefit analysis by mesolimbic dopamine. Psychopharmacology (Berl.).

[26] Salamone JD, Correa M (2012). The mysterious motivational functions of mesolimbic dopamine. Neuron.

[27] Chong, T. T.-J. *et al*. Neurocomputational Mechanisms Underlying Valuation of Effort Costs. *Plos Biol*. (in press). 10.1371/journal.pbio.1002598 PMC5325181 28234892

[28] Le Heron, C., Apps, M. a. J. & Husain, M. The anatomy of apathy: a neurocognitive framework for amotivated behavior. *Neuropsychologia*10.1016/j.neuropsychologia.2017.07.003 (2017). 10.1016/j.neuropsychologia.2017.07.003 PMC6200857 28689673

[29] Ang Y-S, Lockwood P, Apps MAJ, Muhammed K, Husain M (2017). Distinct Subtypes of Apathy Revealed by the Apathy Motivation Index. PLOS ONE.

[30] Marin, R. Apathy: Concept, Syndrome, Neural Mechanisms, and Treatment. in **1**, 304–314 (1996). 10.1053/SCNP00100304 10320433

[31] Radakovic R, Abrahams S (2014). Developing a new apathy measurement scale: Dimensional Apathy Scale. Psychiatry Res..

[32] Levy Rb, Dubois B (2006). Apathy and the functional anatomy of the prefrontal cortex-basal ganglia circuits. Cereb. Cortex.

[33] Chow TW (2009). Apathy Symptom Profile and Behavioral Associations in Frontotemporal Dementia vs. Alzheimer’s Disease. Arch. Neurol..

[34] Dermody N (2016). Uncovering the neural bases of cognitive and affective empathy deficits in Alzheimer’s disease and the behavioral-variant of frontotemporal dementia. J. Alzheimers Dis..

[35] Rascovsky K (2011). Sensitivity of revised diagnostic criteria for the behavioural variant of frontotemporal dementia. Brain.

[36] Meffert H, Gazzola V, Boer JA, den, Bartels AAJ, Keysers C (2013). Reduced spontaneous but relatively normal deliberate vicarious representations in psychopathy. Brain.

[37] Decety J, Skelly LR, Kiehl KA (2013). Brain response to empathy-eliciting scenarios involving pain in incarcerated individuals with psychopathy. JAMA Psychiatry.

[38] Lockwood PL (2013). Association of callous traits with reduced neural response to others’ pain in children with conduct problems. Curr. Biol. CB.

[39] Lockwood PL (2017). Prosocial apathy for helping others when effort is required. Nat. Hum. Behav..

[40] Reniers RLEP, Corcoran R, Drake R, Shryane NM, Völlm Ba (2011). The QCAE: a Questionnaire of Cognitive and Affective Empathy. J. Pers. Assess..

[41] Smith RL, Ager JW, Williams DL (1992). Suppressor Variables in Multiple Regression/Correlation. Educ. Psychol. Meas..

[42] Seara-Cardoso A, Sebastian CL, Viding E, Roiser JP (2016). Affective resonance in response to others’ emotional faces varies with affective ratings and psychopathic traits in amygdala and anterior insula. Soc. Neurosci..

[43] Seara-Cardoso A, Dolberg H, Neumann C, Roiser JP, Viding E (2013). Empathy, morality and psychopathic traits in women. Personal. Individ. Differ..

[44] Seara-cardoso A, Neumann C, Roiser J, Mccrory E, Viding E (2012). Investigating associations between empathy, morality and psychopathic personality traits in the general population. Personal. Individ. Differ..

[45] Cattell RB (1966). The scree test for the number of factors. Multivar. Behav. Res..

[46] Cameron, C. D., Hutcherson, C., Ferguson, A. M., Inzlicht, M. & Scheffer, J. A. Empathy is a choice: People are empathy misers because they are cognitive misers. (2017).

[47] Lockwood PL, Seara-Cardoso A, Viding E (2014). Emotion regulation moderates the association between empathy and prosocial behavior. PloS One.

[48] Besel LDS, Yuille JC (2010). Individual differences in empathy: The role of facial expression recognition. Personal. Individ. Differ..

[49] Gery I, Miljkovitch R, Berthoz S, Soussignan R (2009). Empathy and recognition of facial expressions of emotion in sex offenders, non-sex offenders and normal controls. Psychiatry Res..

[50] Martin RA, Berry GE, Dobranski T, Horne M, Dodgson PG (1996). Emotion perception threshold: Individual differences in emotional sensitivity. J. Res. Personal..

[51] Kanske P, Böckler A, Trautwein F-M, Parianen Lesemann FH, Singer T (2016). Are strong empathizers better mentalizers? Evidence for independence and interaction between the routes of social cognition. Soc. Cogn. Affect. Neurosci..

[52] Schwenck C (2012). Empathy in children with autism and conduct disorder: group-specific profiles and developmental aspects. J. Child Psychol. Psychiatry.

[53] Jones AP, Happé FGE, Gilbert F, Burnett S, Viding E (2010). Feeling, caring, knowing: different types of empathy deficit in boys with psychopathic tendencies and autism spectrum disorder. J. Child Psychol. Psychiatry.

[54] Lamm C, Bukowski H, Silani G (2016). From shared to distinct self–other representations in empathy: evidence from neurotypical function and socio-cognitive disorders. Phil Trans R Soc B.

[55] Crockett MJ, Kurth-nelson Z, Siegel JZ, Dayan P, Dolan RJ (2015). Harm to others outweighs harm to self in moral decision making. Proc. Natl. Acad. Sci..

[56] Bonnelle V (2015). Characterization of reward and effort mechanisms in apathy. J Physiol. Paris.

[57] Hartmann MN (2015). Apathy but not diminished expression in schizophrenia is associated with discounting of monetary rewards by physical effort. Schizophr. Bull..

[58] Davis M (1983). Measuring individual differences I empathy: Evidence for a multidimensional approach. J. Pers. Soc. Psychol..

[59] Hogan R (1969). Development of an empathy scale. J. Consult. Clin. Psychol..

[60] Mehrabian A, Epstein N (1972). A measure of emotional empathy. J. Pers..

[61] Baron-Cohen S, Wheelwright S (2004). The empathy quotient: an investigation of adults with Asperger syndrome or high functioning autism, and normal sex differences. J. Autism Dev. Disord..

[62] Worthington RL, Whittaker TA (2006). Scale Development Research A Content Analysis and Recommendations for Best Practices. Couns. Psychol..

[63] Ali F, Amorim IS, Chamorro-Premuzic T (2009). Empathy deficits and trait emotional intelligence in psychopathy and Machiavellianism. Personal. Individ. Differ..

[64] Aifanti, N., Papachristou, C. & Delopoulos, A. The MUG facial expression database. in 1–4 (IEEE, 2010).

[65] Ekman P (1993). Facial expression and emotion. Am. Psychol..

[66] Limbrecht-Ecklundt, K. *et al*. The effect of forced choice on facial emotion recognition: a comparison to open verbal classification of emotion labels. *GMS Psycho-Soc.-Med*. **10** (2013). 10.3205/psm000094 PMC3687244 23798981

[67] McKee SP, Klein SA, Teller DY (1985). Statistical properties of forced-choice psychometric functions: Implications of probit analysis. Percept. Psychophys..

[68] Russell JA (1993). Forced-choice response format in the study of facial expression. Motiv. Emot..

[69] Benjamini, Y. & Hochberg, Y. Controlling the false discovery rate: a practical and powerful approach to multiple testing. *J. R. Stat. Soc. Ser. B Methodol*. 289–300 (1995).

[70] Muthen, L. K. & Muthen, B. O. Mplus [computer software]. *Los Angel. CA Muthén Muthén* (1998).

