# Supplementary material for: Individual differences in empathy are associated with apathy-motivation
Source: Sci Rep. 2017 Dec 11;7:17293. doi: 10.1038/s41598-017-17415-w (PMC5725487; doi:10.1038/s41598-017-17415-w)
Supplement: Supplementary file 1 — Supplementary Information [file 41598_2017_17415_MOESM1_ESM.pdf]

Individual differences in empathy are associated with apathy-motivation

Patricia L. Lockwood, Yuen-Siang Ang, Masud Husain, Molly J. Crockett

Supplementary Information

Supplementary Tables

Table S1: Descriptive statistics for Sample 1.

	N	Mean (S.D.)	Range	Skewness	Kurtosis
**Age**	375	30.5 (11.2)	16 – 74	1.477	1.989
**AMI**					
Behavioural Apathy	378	1.60 (0.76)	0 – 3.67	0.321	-0.303
Social Apathy	378	1.76 (0.76)	0 – 4.00	0.361	-0.312
Emotional Apathy	378	1.10 (0.65)	0 – 3.17	0.802	0.468
Total	378	1.49 (0.48)	0.22 – 3.17	0.455	0.535
**QCAE**					
Cognitive Empathy	378	56.4 (9.4)	21 – 76	-0.405	0.401
Affective Empathy	378	32.3 (5.7)	13 – 47	-0.241	0.028
Total	378	88.7 (12.8)	34 – 122	-0.399	0.877

Table S2: Descriptive statistics for Sample 2.

	N	Mean (S.D.)	Range	Skewness	Kurtosis
**Age**	197	34.3 (10.7)	20 – 63	0.88	0.00
**AMI**					
Behavioural Apathy	198	1.61 (0.79)	0 – 4.00	0.30	0.31
Social Apathy	198	1.94 (0.72)	0.17 – 3.67	-0.02	-0.36
Emotional Apathy	198	1.09 (0.65)	0 – 3.50	0.86	0.93
Total	198	1.54 (0.51)	0.28 – 3.28	0.38	0.56
**QCAE**					
Cognitive Empathy	198	56.7 (8.7)	27 – 76	-0.15	0.32
Affective Empathy	198	33.0 (6.1)	16 – 48	-0.29	0.15
Total	198	89.7 (12.7)	56 – 124	0.12	-0.19
**State empathy ratings**					
Happy	198	7.48 (1.26)	3.49 – 9.97	-0.32	-0.28
Disgust	198	3.49 (1.01)	1.05 – 5.94	-0.09	-0.64
Angry	198	3.66 (0.96)	1.06 – 6.22	-0.21	-0.26
Sad	198	3.51 (0.89)	0.98 – 5.65	-0.03	-0.52
Fear	198	3.90 (0.98)	0.98 – 6.48	-0.12	-0.22
**Emotion recognition accuracy**					
Happy	198	98.5 (3.5)	85-100	-2.24	4.43
Disgust	198	76.9 (17.8)	15 – 100	-1.26	1.55
Angry	198	70.6 (20.2)	0 – 100	-0.95	0.76

Sad	198	82.1 (15.0)	31 – 100	-1.00	0.87
Fear	198	88.3 (12.1)	46 – 100	-1.20	1.09
Total	198	84.6 (7.5)	62 – 97	-0.86	0.56

Supplementary Results

Bivariate associations between empathy and apathy in Sample 1

Cognitive empathy was significantly negatively correlated with *behavioural apathy* (r = -.25, p < .001), suggesting that those high in cognitive empathy are more behaviourally motivated. However, there was no significant association between affective empathy and behavioural apathy (r = .10, p > .06). Both *cognitive empathy* and *affective empathy* were significantly negatively associated with *social apathy* (r = -.26, p < .001 and r = -.16, p = .003 respectively). Finally, *cognitive* and *affective empathy* were significantly negatively associated with *emotional apathy* (r = -.36, p < .001 and r = -.61, p < .001) (**Table S3**).

Table S3 I Bivariate associations between domains of apathy and empathy

	AMI-Behavioural apathy	AMI - Social apathy	AMI - Emotional apathy	QCAE - Peripheral responsivity score	QCAE - Perspective-taking score	QCAE - Online simulation score	QCAE - Emotion contagion score	QCAE - Proximal responsivity score	QCAE - Cognitive empathy score
AMI - Social apathy	.337**								
AMI - Emotional apathy	-0.034	.144**							
Peripheral responsivity score	0.029	-.108*	-.395**						
Perspective-taking score	-.260**	-.260**	-.197**	.208**					
Online simulation score	-.178**	-.198**	-.448**	.291**	.556**				
Emotion contagion score	.231**	-0.007	-.411**	.247**	0.041	.178**			
Proximal responsivity score	-0.05	-.253**	-.596**	.442**	.402**	.507**	.474**		
Cognitive empathy score	-.251**	-.262**	-.355**	.279**	.899**	.863**	.119*	.510**	
Emotional empathy score	0.097	-.155**	-.605**	.723**	.275**	.417**	.761**	.824**	.387**

** Correlation is significant at the 0.01 level (2-tailed).

* Correlation is significant at the 0.05 level (2-tailed).

Pearson partial correlation coefficients are reported (2-tailed). AMI = Apathy-Motivation Index. QCAE = Questionnaire of Cognitive and Affective Empathy

Replication of correlations and regressions between apathy and empathy domains

We sought to replicate our findings in an independent sample of healthy people (n=198) who also completed a task to assess state affective empathy and emotion recognition (see Experimental Procedures for participant demographics). We found that all previously reported bivariate correlations replicated the findings of the first study, with the exception that affective empathy was no longer significantly associated with social apathy (r = -.10, p = .15) (see **Table S4**).

Table S4 l Replication of associations between domains of empathy and apathy-motivation

	AMI-Behavioural apathy	AMI - Social apathy	AMI - Emotional apathy	QCAE - Peripheral responsivity score	QCAE - Perspective-taking score	QCAE - Online simulation score	QCAE - Emotion contagion score	QCAE - Proximal responsivity score	QCAE - Cognitive empathy score
AMI - Social apathy	.451**								
AMI - Emotional apathy	0.097	.201**							
Peripheral responsivity score	-.389**	-.422**	-.225**						
Perspective-taking score	-.239**	-.236**	-.413**	.559**					
Online simulation score	0.104	-0.035	-.505**	0.101	.292**				
Emotion contagion score	0.022	0.008	-.334**	.229**	.386**	.400**			
Proximal responsivity score	-0.138	-.230**	-.549**	.409**	.534**	.573**	.525**		
Cognitive empathy score	-.364**	-.383**	-.350**	.908**	.856**	.211**	.339**	.525**	
Emotional empathy score	0	-0.102	-.568**	.296**	.491**	.816**	.781**	.851**	.433**

** Correlation is significant at the 0.01 level (2-tailed).

Pearson partial correlation coefficients are reported (2-tailed). AMI = Apathy-Motivation Index. QCAE = Questionnaire of Cognitive and Affective Empathy.

Bivariate associations between age, empathy and apathy

Bivariate correlations between age and empathy and apathy showed that age was correlated with behavioural apathy (r = -.11, p = .03) and social apathy (r = .14, p = .007) in Sample 1. We therefore controlled for age in all regression analyses. Note that the analyses with and without controlling for age did not significantly change any of the results.
